# Uptake of intermittent preventive treatment and pregnancy outcomes: health facilities and community surveys in Chókwè district, southern Mozambique

**DOI:** 10.1186/s12936-018-2255-z

**Published:** 2018-03-12

**Authors:** Paulo Arnaldo, Eduard Rovira-Vallbona, Jerónimo S. Langa, Crizolgo Salvador, Pieter Guetens, Driss Chiheb, Bernardete Xavier, Luc Kestens, Sónia M. Enosse, Anna Rosanas-Urgell

**Affiliations:** 1 Department of Biomedical Sciences, Institute of Tropical Medicine Antwerp, Antwerp, Belgium; 2 Plataforma de Parasitologia Molecular, Instituto Nacional de Saúde, Maputo, Mozambique; 3 University of Antwerp, Antwerp, Belgium

**Keywords:** Malaria, Pregnancy, IPTp-SP, Coverage, Risk factors, Mozambique

## Abstract

**Background:**

Malaria in pregnancy leads to serious adverse effects on the mother and the child and accounts for 75,000–200,000 infant deaths every year. Currently, the World Health Organization recommends intermittent preventive treatment of malaria in pregnancy (IPTp) with sulfadoxine–pyrimethamine (SP) at each scheduled antenatal care (ANC) visit. This study aimed to assess IPTp-SP coverage in mothers delivering in health facilities and at the community. In addition, factors associated with low IPTp-SP uptake and malaria adverse outcomes in pregnancy were investigated.

**Methods:**

A community and a health facility-based surveys were conducted in mothers delivering in Chókwè district, southern Mozambique. Social-demographic data, malaria prevention practices and obstetric history were recorded through self-report and antenatal records. For women delivering at health facilities, a clinical examination of mother and child was performed, and malaria infection at delivery was determined by rapid diagnostic test, microscopy, quantitative PCR and placental histology.

**Results:**

Of 1141 participants, 46.6, 30.2, 13.5 and 9.6% reported taking ≥ 3, two, one and none SP doses, respectively. Low IPTp uptake (< 3 doses) was associated with non-institutional deliveries (AOR = 2.9, P < 0.001), first ANC visit after week 28 (AOR = 5.4, P < 0.001), low awareness of IPTp-SP (AOR = 1.6, P < 0.002) and having no or only primary education (AOR = 1.3, P = 0.041). The overall prevalence of maternal malaria (peripheral and/or placental) was 16.8% and was higher among women from rural areas compared to those from urban areas (AOR = 1.9, P < 0.001). Younger age (< 20 years; AOR = 1.6, P = 0.042) and living in rural areas (AOR = 1.9, P < 0.001) were predictors of maternal malaria at delivery. Being primigravidae (AOR = 2.2, P = 0.023) and preterm delivery (AOR = 2.6, P < 0.001) predicted low birth weight while younger age was also associated with premature delivery (AOR = 1.4, P = 0.031).

**Conclusion:**

The coverage for two and ≥ 3 doses of IPTp-SP is moderately higher than estimates from routine health facility records in Gaza province in 2015. However, this is still far below the national target of 80% for ≥ 3 doses. Ongoing campaigns aiming to increase the use of malaria prevention strategies during pregnancy should particularly target rural populations, increasing IPTp-SP knowledge, stimulate early visits to ANC, improve access to health services and the quality of the service provided.

**Electronic supplementary material:**

The online version of this article (10.1186/s12936-018-2255-z) contains supplementary material, which is available to authorized users.

## Background

Although the global burden of malaria has been considerably reduced over recent years, it remains a major public health problem in sub-Saharan Africa (SSA), where 190 million cases and 394,680 deaths occurred in 2015 [1]. It is estimated that in the SSA region, 32 million pregnant women are at risk of acquiring malaria in pregnancy (MiP) every year [2, 3].

MiP is associated with increased risk of both maternal and neonatal adverse outcomes including maternal anaemia (which leads to increased maternal mortality), delivery of low birth weight (LBW) infants, premature delivery, stillbirth and increased perinatal and infant mortality [4, 5]. Women in their first pregnancy are at high risk of infection due to lack of specific immunity against the *Plasmodium falciparum* variant surface antigen VAR2csa, that mediates specific sequestration of parasites to placental tissue [6, 7].

To prevent MiP in areas with moderate to high malaria transmission the World Health Organization (WHO) recommends the use of insecticide-treated nets (ITNs) and intermittent preventive treatment in pregnancy (IPTp) with at least three doses of sulfadoxine–pyrimethamine (SP), administered at scheduled antenatal care (ANC) visits regardless of the presence of parasites and signs of malaria [8]. Despite the fact that IPTp-SP has been rolled out for many years in SSA countries, several studies in the region report a low IPTp-SP coverage [9, 10]. In 2015, only 50% of women in 36 reporting countries in the African region received two SP doses, while only 31% received ≥ 3 doses [2]. The major factors that have been associated with low IPTp-SP uptake include the number and timing of ANC visits, the lack of knowledge of MiP adverse consequences [11], systemic factors such as lack of clear policies and guidelines, as well as insufficient training, supervision, and quality assurance at the health facility level [10, 12–14] and drug stock-outs [15]. In addition, women that have non-institutional deliveries (deliveries at home) may be more likely to benefit less from health care including prenatal consultations and hence, receive less IPTp-SP doses [16, 17].

In Mozambique, where malaria is endemic and transmission is perennial, MiP is the most important cause of maternal death and contributes to the high overall maternal mortality rates (408 of maternal mortality per 100,000 births in 2011) [18] and delivery of LBW infants (4.3% overall in 2016) observed in the country [19]. IPTp-SP was first implemented in the country in 2006 delivered free of charge to all pregnant women under directly-observed treatment (DOT) [20]. In 2014, the national guidelines were updated and implemented countrywide to adjust to the current ≥ 3 SP-dose WHO recommendation [21]. Although the collection of robust data on IPTp coverage is essential to monitor, evaluate and further improve the currently implemented MiP interventions, in Mozambique IPTp coverage available data is based mainly on routine annual reports from health centres (often lacking completeness and accuracy) [21], household surveys (HH) of HIV and malaria indicators at the province level [22] and retrospective studies (which do not reflect the current situation) [15].

In 2015, a national HH survey with data collection at the provincial level, reported an IPTp-SP country coverage of 51.4% for one dose, 34.2% for two doses and 22.4% for ≥ 3 doses [22]. This study was conducted to determine the coverage of IPTp-SP uptake in Chókwè district (Gaza Province), where malaria prevalence in the general population was reported at 32.5% in 2016 (Chókwè district health services 2016, unpublished data), at the time of implementation of the new WHO recommendation of IPTp. Importantly, the study collected information on factors potentially related to low IPTp-SP uptake both in health facilities and at the community level (among women with non-institutional deliveries). In addition, on these mothers delivering at the health facility, factors associated with malaria infection at delivery and adverse pregnancy outcomes were also investigated.

## Methods

### Study site

**Fig. 1:**
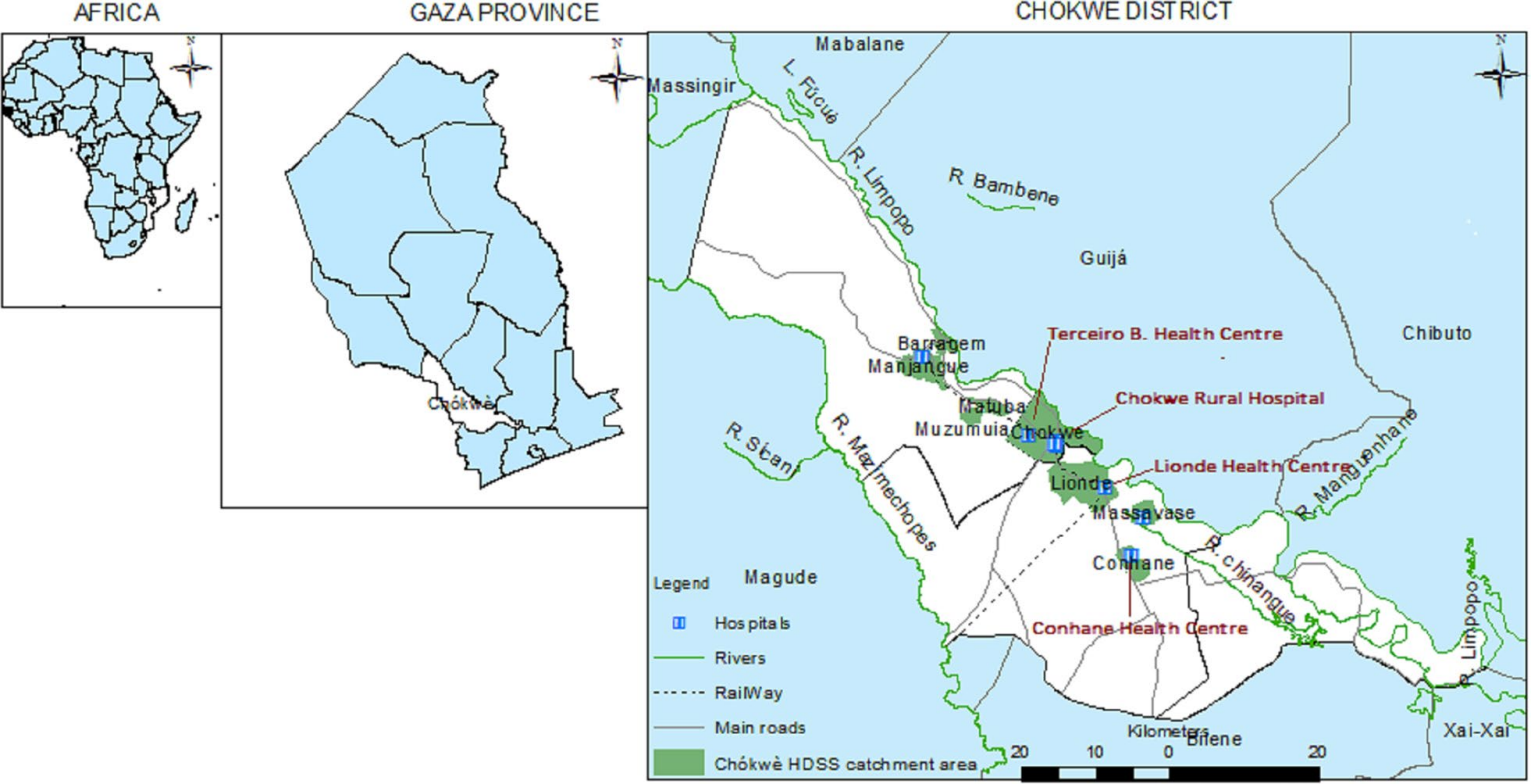
Map of Chókwè district showing the Chókwè HDSS catchment area and study Health Centres (Source: Chókwè HDSS 2014)

This study was conducted in the Chókwè district between June 2014 and June 2015. Chókwè is located in Gaza Province along the Limpopo River in the southern region of Mozambique, approximately 220 kilometres northwest of Maputo, the capital city of the country. The district has an area of 2466 km^2^ and an estimated population of 214,183 inhabitants [23]. Chókwè district includes four administrative areas: Chókwè, Liónde, Macarretane and Xilembene (Fig. 1). The main economic activities of the district population are subsistence farming, large rice productions supported by major irrigation systems, livestock keeping and small business. Malaria, which is mostly attributable to *P. falciparum*, is endemic in this area with the majority of cases occurring during the rainy season from November to April.

A continuous health and demographic surveillance system (HDSS)—including 135,616 habitants (63.3% of the district population) and occupying an area of approximately 600 km^2^ within a 25 km radius of Chókwè City is run by the “Centro de Investigação e Treino em Saúde de Chókwè”, a clinical research centre affiliated with the National Institute of Health—Ministry of Health of Mozambique. The HDSS catchment area includes fifteen villages, eight of which belong to the Chókwè Municipality (classified as urban) and seven to Liónde and Macarretane (classified as rural). Data routinely registered in the HDSS includes migrations, pregnancies, births and deaths (Bonzela et al. pers.comm.).

### Study design and population

Although during the last 10 years Mozambique has achieved significant progress in the coverage of institutional deliveries, an important proportion of these still occur mainly in rural communities [18, 19, 22]. Therefore, to obtain accurate data on IPTp coverage in the area under HDSS surveillance, a community and health facility based surveys were conducted. The community survey included women with non-institutional deliveries during the study period, while the health facility survey included women delivering at (i) Chókwè Hospital, (ii) Peripheral Health Centre of Terceiro Bairro, (iii) Peripheral Health Centre of Liónde and (iv) Peripheral Health Centre of Conhane, all located within the Chókwè HDSS catchment area (Fig. 1). Chókwè Hospital is located in urban area (Chókwè municipality) and is the reference hospital providing assistance for all Chókwè district population and neighbouring districts.

Women were enrolled in the study if they fulfilled the following inclusion criteria: (i) aged between 15 and 48 years, (ii) having a singleton delivery, and (iii) being a permanent resident of the area under HDSS surveillance in the Chókwè district. HIV positive women receiving antiretroviral treatment or prophylactic treatment with co-trimoxazole were excluded from the study, since IPTp-SP is not recommended in these women due to potential adverse drug reactions [24]. Written informed consents were obtained from all women before enrolment into the study.

### Sample size

In Mozambique, WHO’s recommendation of at least three doses of IPTp-SP during pregnancy was implemented in 2014. Therefore, the sample size calculation was based on the estimate of  the proportion of pregnant women receiving three or more IPTp-SP in Gaza province. This was approximately 28% in 2014 [21]. With a margin of error of ± 4% using an alpha type-1 error of 5%, at least 1038 delivering women during the study period were estimated to be included.

### Data collection and study procedures

Data were collected by trained midwives and HDSS health-workers who were specifically trained for this study. A structured questionnaire in Portuguese and local Changana language was administrated to document socio-demographic data including age, residence, marital status, education, occupation, knowledge of IPTp and use of ITN. Antenatal data were obtained from the mother’s antenatal card and included parity, gestational age at the first ANC, and timing of IPTp-SP doses. Women with non-institutional deliveries were identified through the HDSS by comparing births registered in the database and hospital registers, and the interviews were conducted within 3 months after the date of delivery to minimize recall bias.

In the health facility survey, parturient women were examined by a hospital clinician. Axillary body temperature was recorded and 3 ml of venous blood sample was collected immediately after delivery in ethylene diamine tetra acetic acid (EDTA) containing tubes. Peripheral venous blood samples were used to assess hemoglobin levels (HemoCue 301, Angelholm, Sweden), *P. falciparum* infection using RDT (SD Bioline Malaria Antigen Pf, Standard Diagnostic Inc, South Korea) and to prepare blood slides and samples for posterior diagnosis by light microscopy and quantitative polymerase chain reaction (qPCR), respectively.

Newborn’s birth weight and gestational age were measured within 24 h after delivery: birth weight using a digital scale (Soehnle professional, Soehnle Industrial Solutions GmbH; Germany) and gestational age based on last normal menstrual period; in case of uncertainty, it was estimated with the Ballard score maturational assessment by trained midwives [25]. Placental tissue samples were collected from the maternal side of the placenta with approximately 2 cm × 2 cm in length and width, and 1 cm in depth and immediately placed in 10% neutral buffer formalin and stored at 2–8 °C until processed.

### Laboratory procedures

Thick and thin smears were prepared and stained with 5% Giemsa for 25 min and examined for malaria parasites by standard microscopy [26]. Slides were examined by two independent microscopists from the National Malaria Reference Laboratory using light microscopy at 100× magnification for the presence of malaria parasites. Parasite density was estimated by counting the number of asexual parasites per 500 white blood cells (WBCs), and parasites per µL calculated assuming a WBC count of 8000 cells per µL of whole blood. A slide was considered to be negative if no parasites were seen after review of 1000 WBCs. In case of discrepant results, the slide was read by a third microscopist and the mean of the two closest reads was used. External quality control with 10% of slides was performed by a fourth experienced reader at the Malariology Unity Laboratory, Institute of Tropical Medicine in Antwerp, Belgium.

Molecular detection of *P. falciparum* infections was performed by qPCR. Briefly, DNA was extracted from 200 μL of erythrocyte pellet with QIAamp 96 DNA blood kit (Qiagen, Germany), and eluted in 200 μL of water. Five microliters of DNA were used for qPCR analysis targeting *P. falciparum var* gene acidic terminal sequence (*var* ATS, ~ 59 copies per genome) as previously described [27]. Parasite densities were obtained by interpolating cycle thresholds (Ct) from a standard curve of infected erythrocytes diluted in whole blood (from 100,000 to 0.01 parasites/μL). Samples with Ct values ≤ 38.5 Ct were considered positive. The limit of detection was 0.04 parasite/µL.

Placental tissue preparation and histological examination was performed at the Pathology laboratory of the Maputo Central Hospital (Hospital Central de Maputo-HCM) as described elsewhere [28]. Two trained-independent microscopists read the slides, discrepant results were reviewed by a third microscopist and a consensus result was determined. External quality control was performed at Barcelona Institute for Global Health (ISGlobal) by a fourth experienced reader for 10% of slides.

### Definitions

The following definitions were used: (a) fever: axillary temperature ≥ 37.5 °C; (b) moderate and severe anaemia: Hb < 11 g/dL and Hb < 8 g/dL respectively; (c) LBW: < 2,500 g; (d) preterm delivery: < 37 weeks of completed gestation. Gravidity was categorized into primigravidae (women in their first pregnancy) and multigravidae (women in their second or more pregnancies). Placental infection was classified according to the histopathology results as: (i) uninfected, no parasites or pigment present; (ii) acute infection, parasites present with no pigment in monocytes or fibrin; (iii) chronic infection, parasites present in erythrocytes with pigment and (iv) past infection, no parasites, pigment confined to fibrin or cells within fibrin indicating past infection [29].

### Ethics statement

This study was approved by the National Bioethics Committees of Mozambique (CNBS) (IRB00002657), the Institute of Tropical Medicine (ITM) Institutional Review Board (IRBAB/ac/059) and the University of Antwerp (IRB-B300201421228). All procedures were carried out in accordance with the Helsinki Declaration as revised in 2013. Administrative approval to conduct the study was obtained from the local health facilities and the Ministry of Health of Mozambique. Informed consent was obtained at recruitment from all study participants or their representatives.

### Statistical analysis

Data from all study forms were double entered and checked for unusual values and inconsistencies between fields using OpenClínica v.3.3 (USA), and then exported to STATA version 14.1 (Stata Corp, College Station, TX, USA) for analysis. For categorical variables, descriptive analysis was performed and the data summarized in proportions and frequency tables. Means with their respective standard deviations and medians with interquartile ranges were used to summarize continuous variables. Kruskal–Wallis rank test was used to compare medians and interquartile ranges (IQRs) of continuous variables, and Chi square test or Fisher exact test for categorical variables. Univariate analysis was performed to analyse factors associated with low IPTp-SP uptake (< 3 doses), maternal malaria infection and density, LBW and preterm deliveries. Explanatory variables with *P *< 0.20 in the univariate analysis, were included in the multivariable regression analysis. Crude odds ratios (OR), adjusted odds ratios (AOR) and 95% confidence intervals are reported with *P* values < 0.05 considered statistically significant.

## Results

### Characteristics of the study population (community and health facility surveys)

During the study period, 3854 deliveries were registered in the HDSS catchment area. Of these, 1330 (34.5%) women were screened and 14.2% (189/1330) were excluded due to HIV positivity, non-singleton birth or living outside the Chókwè district. In total, 1141 women were interviewed and included in the study, from which 80.1% (914/1141) were recruited in maternity hospitals and 19.9% (227/1141) in the community (non-institutional deliveries).

Demographic characteristics of study participants are shown in Table 1 (first and second columns). More than two-thirds (70.6%) of the women included in the study lived in Chókwè municipality (urban area), compared to 29.4% living in the rural areas of Lionde and Macarretane. Of the 227 women who had non-institutional deliveries, 67.0% were from rural areas, and 33.0% from urban area (P < 0.001) (Additional file 1: Table S1).

**Table 1:** Characteristics of participants by place of delivery and association with non-institutional delivery

Variable	N	Place of delivery		OR 95% CI	P value	AOR 95% CI	P value
		Health facility (n = 914)	Home (n = 227)				
		n [%]	n [%]				
Age							
< 20	380	345 [37.8]	35 [15.4]	Ref.		Ref.	
≥ 20	761	569 [62.2]	192 [84.6]	*3.3 [2.2–4.8]*	*<* *0.001*	1.3 [0.5–2.2]	0.712
Gravidity							
Primigravidae (1)	427	395 [43.2]	32 [14.1]	Ref.		Ref.	
Multigravidae (≥ 2)	714	519 [56.8]	195 [85.9]	*4.6 [3.1–6.8]*		*2.3 [1.2–4.6]*	*0.017*
Place of residence							
Urban	806	731 [80.0]	75 [33.0]	Ref.		Ref.	
Rural	335	183 [20.0]	152 [67.0]	*8.1 [5.9–11.2]*	*<* *0.001*	*3.2 [2.1–4.9]*	*<* *0.001*
Marital status							
Single	294	236 [25.8]	58 [25.5]	0.9 [0.7–1.3]	0.934	–	
Married/cohabiting	847	678 [7402]	169 [74.5]	Ref.			
Education							
None/primary school	674	477 [52.2]	197 [86.8]	*6.0 [4.0–9.0]*	*<* *0.001*	*2.3 [1.4–3.7]*	*<* *0.001*
Secondary/high school	467	437 [47.8]	30 [13.2]	Ref.		Ref.	
Occupation							
Unemployed	882	802 [87.6]	80 [35.2]	1.4 [0.5–3.7]	0.414	1.3 [0.5–3.6]	0.503
Agro-livestock	180	38 [4.2]	142 [62.6]	*55.3 [20–146]*	*<* *0.001*	*21.2 [7.6–59.1]*	*<* *0.001*
Employed/self employed	79	74 [8.1]	5 [2.2]	Ref.		Ref.	
Bed net use							
Yes	1055	844 [92.3]	211 [92.9]	Ref.			
No	86	70 [7.7]	16 [7.1]	0.9 [0.5–1.6]	0.755	–	
Timing of first ANC visit (weeks)							
< 28	1055	841 [92.0]	214 [94.3]	Ref.			
≥ 28	86	73 [8.0]	13 [5.7]	0.6 [0.3–1.2]	0.251	–	
Reported malaria pregnancy							
No	1001	810 [88.6]	191 [84.1]	Ref.		Ref.	
Yes	140	104 [11.3]	36 [15.9]	1.4 [0.9–2.2]	0.067	1.3 [0.7–2.2]	0.338
Ever heard about IPTp-SP							
Yes	251	713 [78.0]	177 [77.9]	Ref.			
No	890	201 [22.0]	50 [22.1]	1.0 [0.7–1.4]	0.991	–	

*CI* confidence interval, *ANC* antenatal care, *OR* odds ratio, *AOR* adjusted odds ratio, *Ref.* reference category

Significant P values are presented in italics

In this study, 92.5% (1055/1141) of women had their first ANC visit before 28 weeks (or during the first or second trimester of gestation). The majority of women starting ANC visit in the third trimester of gestation lived in urban areas 82.7% (71/86), 70.9% (61/86) had no formal education, 81.4% (70/86) were multigravidae, and 59.3% (51/86) delivered at Chókwè hospital, while 25.6% (22/86) delivered at peripheral health centres and 15.1% (13/86) had non-institutional deliveries (P = 0.011).

Compared to women delivering in health facilities, women with non-institutional deliveries (Table 1) showed a higher proportion of multigravida (85.9% vs 56.8%, P = 0.017), were more likely to live in rural areas (67.0% vs 20.0%, P < 0.001), had none or primary education only (86.8% vs 52.2%, P < 0.001) and mainly worked in agriculture or livestock (62.6% vs 4.2%, P < 0.001). When women were questioned (open question) about the main reasons for not delivering at health facility their answers were: lack of transport (55.5%), unexpected delivery date (40.9%), no nurse present at the hospital (1.3%), lack of electricity in the maternity board (1.8%), and desired to deliver at home (0.9%).

### IPTp-SP coverage and factors associated with low uptake

Overall, 532 (46.6%) women participating in the study received ≥ 3 doses of IPTp-SP, 345 (30.2%) received two doses, 154 (13.5%) received one and 110 (9.6%) received no IPTp-SP at all. Analysis of factors associated with low IPTp-SP uptake is presented in Table 2. In the univariate analysis, non-institutional delivery (OR = 3.5, P < 0.001), place of residence (OR = 1.7, P < 0.001), having no or only primary education (OR = 1.8, P < 0.001), working in agriculture (OR = 2.9, P < 0.001), having the first ANC visit after 28 weeks of gestation (OR = 4.9, P < 0.001) and poor knowledge about IPTp (OR = 1.6, P < 0.001), were associated with uptake of < 3 IPTp-SP doses, while being aged < 20 year old (OR = 0.7, P = 0.018) and primigravidae (OR = 0.7, P = 0.002) were associated with uptake of ≥ 3 IPTp-SP doses. However, in the multivariate analysis women with non-institutional delivery (AOR = 2.9, P < 0.001), having no or only primary education (AOR = 1.3, P = 0.041), having the first ANC visit after 28 weeks of gestation (AOR = 5.4, P < 0.001) and poor knowledge about IPTp-SP (AOR = 1.6, P < 0.001) remained associated with low IPTp-SP uptake (< 3 doses).

**Table 2:** Characteristics of participants according to IPTp-SP dose and factors associations with low (< 3 doses) IPTp-SP uptake

Variable	Total [N = 1.141]	IPTp doses				OR 95% CI	P value	AOR 95% CI	P value
		None [n = 110]	One [n = 154]	Two [n = 345]	≥ 3 [n = 532]				
	n [%]	n [%]	n [%]	n [%]	n [%]				
Age (years)									
Median [IQR]	22 [19–28]	25[20–35]	22 [18–28]	22 [19–28]	21 [18–27]				*<* *0.001*
< 20	380 [33.3]	21 [5.5]	57 [15.0]	106 [27.9]	196 [51.6]	*0.7 [0.6–0.9]*	*0.018*	0.9 [0.7–1.4]	0.809
≥ 20	761 [66.7]	89 [11.7]	97 [12.7]	239 [31.4]	336 [44.2]	Ref.		Ref.	
Gravidity									
Median [IQR]	2 [1–4]	3 [2–5]	2 [1–3]	2 [1–4]	2 [1–3]				*<* *0.001*
Primigravidae (1)	427 [37.4]	20 [4.6]	55 [12.9]	127 [29.7]	225 [52.7]	*0.7 [0.5–0.8]*	*0.002*	0.9 [0.6–1.4]	0.864
Multigravidae (≥ 2)	714 [62.6]	90 [12.6]	99 [13.9]	218 [30.5]	307 [43.0]	Ref.		Ref.	
Place of delivery									
Health facility	914 [80.0]	59 [7.3]	115 [12.6]	262 [28.7]	478 [52.3]	Ref.		Ref.	
Non-institutional (home)	227 [20.0]	51 [15.2]	39 [17.2]	83 [37.1]	54 [23.8]	*3.5 [2.5–4.8]*	*<* *0.001*	*2.9 [1.8–4.5]*	*<* *0.001*
Place of residence									
Urban	806 [70.6]	59 [7.3]	107 [13.3]	232 [28.8]	408 [50.6]			Ref.	
Rural	335 [29.4]	51 [15.2]	47 [14.0]	113 [33.8]	124 [37.0]	*1.7 [1.3–2.3]*	*<* *0.001*	1.2 [0.8–1.7]	0.205
Marital status									
Single	294 [74.2]	36 [12.2]	39 [13.3]	91 [31.0]	128 [43.5]	1.2 [0.9–1.5]	0.218	**–**	
Married/cohabiting	847 [25.8]	74 [8.7]	115 [13.6]	254 [30.0]	404 [47.7]	Ref.			
Education									
None/primary school	674 [59.1]	87 [12.9]	107 [15.9]	207 [30.7]	237 [40.5]	*1.8 [1.4–2.3]*	*<* *0.001*	*1.3 [1.0–1.7]*	*0.041*
Secondary/high school	467 [40.9]	23 [4.9]	47 [10.1]	138 [29.6]	259 [55.5]	Ref.		Ref.	
Occupation									
Unemployed	882 [77.3]	65 [7.4]	110 [12.5]	264 [29.9]	443 [50.2]	1.1 [0.6–1.7]	0.776	1.0 [0.6 –1.7]	0.942
Agro-livestock	180 [15.8]	38 [21.1]	31 [17.2]	63 [35.0]	48 [26.7]	*2.9 [1.7–5.1]*	*<* *0.001*	1.1 [0.6–2.2]	0.750
Employed/self employed	79 [6.9]	7 [8.9]	13 [16.4]	18 [22.8]	41 [51.9]	Ref.		Ref.	
Bed net use									
Yes	1055 [92.5]	95 [9.0]	146 [13.8]	316 [30.0]	498 [47.2]	Ref.		Ref.	
No	86 [7.5]	15 [17.5]	8 [9.3]	29 [33.7]	34 [39.5]	1.3 [0.9–2.1]	0.172	1.3 [0.8–2.1]	0.328
Timing of first ANC visit									
< 28 weeks	1055 [92.5]	96 [9.1]	132 [12.5]	309 [29.3]	518 [49.1]	Ref.		Ref.	
≥ 28 weeks	86 [7.5]	14 [16.3]	22 [25.6]	36 [41.9]	14 [16.3]	*4.9 [2.7–8.9]*	*<* *0.001*	*5.4 [2.9–9.8]*	*<* *0.001*
Reported malaria pregnancy									
No	1001 [87.7]	95 [9.5]	131 [13.1]	309 [30.9]	466 [46.5]	Ref.			
Yes	140 [12.3]	15 [10.7]	23 [16.4]	36 [25.7]	66 [47.2]	0.9 [0.7–1.4]	0.896	–	
Ever heard about IPTp-SP									
Yes	251 [22.0]	49 [19.5]	38 [15.1]	70 [27.9]	94 [37.5]	Ref.		Ref.	
No	890 [78.0]	61 [6.9]	116 [13.0]	275 [30.9]	438 [49.2]	*1.6 [1.2–2.2]*	*<* *0.001*	*1.6 [1.2–2.2]*	*0.002*

*IQR* interquartile range, *ANC* antenatal care, *CI* confidence interval, *OR* odds ratio, *AOR* adjusted odds ratio, *Ref.* reference category

Significant P values are presented in italics

### Factors associated with malaria infection at delivery

The overall prevalence of maternal malaria infections at delivery (including peripheral and/or placental infections) was of 16.8% (154/914) of which 35.1% (54/154) had only placental infection, 46.7% (72/154) only peripheral infection and 16.8% (26/154) had both placental and peripheral infections. In the univariate analysis being < 20 years old (OR = 1.3, P = 0.012), living in rural areas (OR = 2.0, P < 0.001) and having had malaria during pregnancy (OR = 1.6, P = 0.039) were associated with increased odds of maternal malaria infection at delivery (Table 3). However, in the multivariate analysis being < 20 years old (AOR = 1.6, P = 0.042) and living in rural areas (AOR = 1.9, P < 0.001) remained significantly associated with maternal malaria infection at delivery. Furthermore, primigravidae women were significantly associated with higher peripheral parasite densities (P = 0.020, Table 4). Of the 914 women delivering at health facilities, 51.8% had moderate anaemia (≥ 8 and < 11 g/dL) and 6.6% had severe anaemia (< 8 g/dL).The overall prevalence of *P. falciparum *peripheral infections at delivery was 10.7% (98/914) by qPCR, 2.7% (25/914) by light microscopy and 2.6% (24/914) by RDT. Among the 98 women with peripheral malaria infection at delivery by qPCR, 76.5% (75/98) were asymptomatic and sub-microscopic, while 21.4% (21/98) had asymptomatic and microscopic infections and only 2.0% (2/98) were symptomatic with sub-microscopic infections. Parasite densities ranged from 0.04 to 80,160 parasites/μL with a median density of 2.3 [0.3-362] parasites⁄μL. The median of parasite density among mothers with asymptomatic infection was 1.9 [0.2-353] parasites/μL, compared to 824 [3.0-1646] parasites/μL of those with symptomatic infection (P=0.410). The median of parasite density in mothers receiving <3 doses of IPTp-SP was 8.4 [0.1-104] parasites/μL compared to 1.8 [0.2-454] parasites/μL in those mothers receiving ≥ 3 doses (P=0.454). Placental malaria infections (PM) by histology were detected in 8.8% (80/914) of women, from which 15.0% (12/80) were active infections and 85.0% (68/80) were past infections. Nearly half of mothers with PM 43.8% (35/80) were <20 years and primigravidae, and 32.5% (26/80) had also peripheral parasitaemia. Among the 80 mothers with PM 6.3% (5/80) did not receive IPTp-SP at all, 16.3% (13/80) received one dose, 27.5% (22/80) received two doses and 50.0% (40/80) received ≥3 doses, and was not significantly different from mothers with no PM (6.6% (8/34) did not receive IPTp-SP, 16.3% (13/834) received one dose, 28.8% (240/834) received two doses and 52.5% (438/834) received ≥3 doses (P=0.784)).

**Table 3:** Factors associated with malaria infection (peripheral and/or placental) among delivering women in the health facility survey (N = 914)

Potential factors	Maternal malaria infection any (peripheral and/or placental)					P value
	N	Infected n [%]	OR 95% CI	P value	AOR 95% CI	
Age (years)						
< 20	345	72 [20.9]	*1.3 [0.9–1.9]*	*0.012*	*1.6 [1.0–2.8]*	*0.042*
≥ 20	569	82 [14.4]	Ref.		Ref.	
Gravidity						
Primigravidae (1)	395	76 [19.2]	1.3 [0.9–1.9]	0.093	0.9 [0.5–1.5]	0.727
Multigravidae (≥ 2)	519	78 [15.0]	Ref.		Ref.	
Residence location						
Urban	731	107 [14.6]	Ref.		Ref.	
Rural	183	47 [25.7]	*2.0 [1.4–2.9]*	*<* *0.001*	*1.9 [1.3–2.9]*	*<* *0.001*
Marital status						
Single	236	44 [18.6]	1.2 [0.8–1.7]	0.393	**–**	**–**
Married/cohabiting	678	110 [16.2]	Ref.			
Education						
None/primary school	477	78 [16.4]	0.9 [0.6–1.3]	0.675	**–**	**–**
Secondary/high school	437	76 [17.4]	Ref.			
Malaria in pregnancy						
No	810	129 [15.9]	Ref.		Ref.	
Yes	104	25 [24.0]	*1.6 [1.0–2.7]*	*0.039*	1.5 [0.9–2.5]	0.093
IPTp-SP receipt						
< 3 doses	436	69 [15.8]	0.8 [0.6–1.2]	0.430	**–**	**–**
≥ 3 doses	478	85 [17.8]	Ref.			
Anemia (< 11 g/dL)^a^						
Yes	470	76 [16.2]	0.9 [0.6–1.3]	0.560	**–**	**–**
No	437	77 [17.6]	Ref.			
Bed net use						
Yes	844	142 [16.8]	Ref.			

*CI* confidence interval, *OR* odds ratio, *AOR* adjusted odds ratio, *Ref.* reference category

^a^Data was unavailable in seven participants (n = 907)

Significant P values are presented in italics

**Table 4:** Analysis of factors associated with higher mean peripheral parasite density among delivering women in the health facility survey

Potential factors	Mean	Coefficient		Coefficient	
	Parasite density ± SD	95% CI	P value	95% CI	P value
Age (years)					
< 20	8276 ± 19,958	*6495 [507–12,483]*	*0.034*	− 2089 [− 10,606 to 6428]	0.627
≥ 20	1781 ± 8980	Ref.		Ref.	
Gravidity					
Primigravidae (1)	9473 ± 21,336	*8952 [3122–14,782]*	*0.003*	*9832 [1572–18,093]*	*0.020*
Multigravidae (≥ 2)	520 ± 1526	Ref.		Ref.	
Residence location					
Urban	4299 ± 14,209	Ref.			
Rural	5178 ± 16,642	878 [− 5394 to 7152]	0.782	**–**	**–**
Education					
None/primary school	5742 ± 17,126	2176 [− 3893 to 8247]	0.478	**–**	**–**
Secondary/high school	3565 ± 12,937	Ref.			
IPTp-SP receipt					
< 3 doses	4991 ± 16,174	− 819 [− 6948 to 5309]	0.791	**–**	**–**
≥ 3 doses	4171 ± 13,767	Ref.			
Bed net use					
Yes	4366 ± 14,784	Ref.			
No	8703 ± 20,521	4337 [− 8323 to 16,997]	0.498	**–**	**–**

*SD* standard deviation, *CI* confidence interval, *Ref.* reference category

Significant P values are presented in italics

### Factors associated with maternal anaemia, LBW infants and premature deliveries

Overall, 7.7% (70/914) mothers delivered LBW infants, 20.8% (190/914) women had preterm deliveries and 1.1% (10/914) stillbirths. The median birth weight in the study population was 3025 (2800–3400) g, 38.6% (27/70) of LBW infants were pre-term deliveries. The gestational age of women with preterm delivered babies ranged from 22 to 36 weeks with a mean of 34.7 ± 2.25 weeks. Factors associated with LBW and premature delivery were assessed by univariate and multivariate regression analysis (Table 5). Gravidity (AOR = 2.2, P = 0.023) and pre-term delivery (AOR = 2.6, P < 0.001) remained independently associated with LBW in the multivariate analysis. Moreover, receiving < 3 doses of IPTp-SP (OR = 1.3, P = 0.044) predicted premature delivery in the univariate analysis, although this association was not significant after adjusting by the other covariates. Only being < 20 years old was independently associated with premature delivery in the multivariate analysis (AOR = 1.4, P = 0.031).

**Table 5:** Factors associated with low birth weight and premature delivery among study participants

Potential factors	N	Low birth weight					Premature delivery				
		BW < 2500 g	OR 95% CI	P value	AOR 95% CI	P value	GA < 37 weeks	OR 95% CI	P value	AOR 95% CI	P value
		n [%]					n [%]				
Age (years)											
< 20	345	39 [11.3]	*2.2 [1.3–3.6]*	*0.002*	1.1 [0.5–2.2]	0.699	83 [24.1]	1.3 [0.9–1.8]	0.058	*1.4 [1.0–1.9]*	*0.031*
≥ 20	569	31 [5.4]	Ref.		Ref.		107 [18.8]	Ref.		Ref.	
Gravidity											
Primigravidae (1)	395	45 [11.4]	*2.5 [1.5–4.2]*	*<* *0.001*	*2.2 [1.1–4.8]*	*0.023*	85 [21.5]	1.1 [0.7–1.4]	0.635	–	
Multigravidae (≥ 2)	519	25 [4.8]	Ref.		Ref.		105 [20.2]	Ref.			
Residence location											
Urban	731	54 [7.4]	Ref.				146 [20.0]	Ref.			
Rural	183	16 [8.7]	1.2 [0.6–2.2]	0.538	–		44 [24.0]	1.2 [0.8–1.8]	0.228	–	
Education											
None/primary school	477	37 [7.8]	1.0 [0.6–1.6]	0.907	–		109 [22.9]	1.3 [0.9–1.7]	0.109	1.3 [0.9–1.8]	0.091
Secondary/high school	437	33 [7.6]	Ref.				81 [18.5]	Ref.		Ref.	
Peripheral infection											
Yes	100	8 [8.0]	1.0 [0.5–2.3]	0.892	–		13 [13.0]	1.1 [0.6–1.7]	0.752	–	
No	814	62 [7.6]	Ref.				177 [21.7]	Ref.			
Placental infection											
Yes	80	10 [12.5]	1.8 [0.9–3.7]	0.093	1.8 [0.8–3.8]	0.101	22 [27.5]	0.7 [0.3–1.3]	0.297	–	
No	834	60 [7.2]	Ref		Ref.		168 [20.1]	Ref.			
IPTp-SP uptake											
< 3 doses	436	38 [8.7]	1.3 [0.8–2.2]	0.252	–		103 [23.6]	*1.3 [1.0–1.9]*	*0.044*	1.3 [0.9–1.8]	0.060
≥ 3 doses	478	32 [6.7]	Ref.				87 [18.2]	Ref.		Ref.	
Premature delivery											
Yes	190	27 [14.2]	*2.6 [1.5–4.3]*	*<* *0.001*	*2.6 [1.5–4.5]*	*<* *0.001*	–			–	
No	724	43 [5.9]	Ref.		Ref.						

*CI* confidence interval, *BW* birth weight, *GA* gestational age, *OR* odds ratio, *AOR* adjusted odds ratio

Significant P values are presented in italics

## Discussion

This study was conducted to evaluated the coverage of IPTp-SP in Chókwè district since the implementation of the new WHO recommendations. Importantly, estimates were assessed both in health facilities and in the community (women with non-institutional deliveries), which allowed us to accurately investigate the factors affecting IPTp uptake under routine circumstances in women with different access to health facilities. Results show that the coverage of ≥ 3 doses of IPTp-SP is of 46.6% in the study population. The coverage of the recommended dosing was higher than estimates from the 2015 HH survey in the Gaza province 37.2% [22], reflecting geographical variations in the coverage of IPTp-SP within the province. Moreover, the current coverage is still far below the national target of 80% of pregnant women [30]. The majority of African countries have adopted a policy of providing ≥ 3 doses of IPTp to pregnant women, however, coverage estimates remain far below global targets [31, 32]. In 2014–2016, the overall percentage of women who received ≥ 3 doses of IPT-SP during pregnancy in sub-Saharan Africa ranged from 13 to 19% [32], while in recent studies, IPTp-SP coverage ranged from 6 to 87.3% [33–35]. Non-institutional deliveries were strongly associated with low IPTp uptake in the study population living in rural areas (67%), while the major reasons for delivering outside health facilities were lack of transport (55%) and unexpected delivery date (40.9%). None or only primary education, late timing of first ANC visit and poor awareness about IPTp were also associated with low IPTp uptake, similar to what has been observed in other sub-Saharan Africa countries [34, 36, 37].

At the beginning of the study, it was hypothesized that women with non-institutional deliveries would be those at higher risk of low IPTp uptake due to lower number or later initiation of ANC visits [16, 17]. Since IPTp-SP in Mozambique is delivered free of charge to pregnant women under DOT during ANC visits, the earlier the ANC visits start, the higher is the chance to receive adequate IPTp-SP dosage. Moreover, early and regular ANC attendance provides time for antenatal health education about malaria preventive strategies during pregnancy. However, in the study population, although the odds of failing to take the recommended IPTp-SP doses was five times higher among women initiating ANC visits during the third trimester of gestation compared to those starting during the first or second trimester, significant differences in time to first ANC visit between women delivering in hospital facilities and with non-institutional deliveries were not observed (94.3% of women with non-institutional deliveries and 92% of women delivering at health facilities -reference and peripheral health facilities- had the first ANC visit during the first or second trimester of pregnancy). Future studies should investigate the proportion of ANC visits occurring during the first trimester of pregnancy, as is recommended by WHO in order to maximize the chances for IPTp-SP uptake [8].

Other studies have shown that barriers to adequate health (quality) care access decrease opportunities to adequate IPTp uptake [10, 38]. Although, no SP stock-outs in the study area have been documented by the MoH in Maputo since 2013 (Chókwè district health services 2016, unpublished data), it is possible that women at ANC visits were not always supplied with IPTp-SP, as has been reported in previous studies in SSA [11, 38].

Awareness about IPTp-SP and education level were also critical factors influencing the uptake of IPTp-SP in Chókwè district, as observed in similar previous studies from other East African countries [36, 39]. These results confirm that health education on IPTp-SP and promoting formal general education beyond primary school will apprise and influence decisions and further increase coverage of the recommended dosing among pregnant women.

The prevalence of maternal *P. falciparum* infection at delivery (defined as peripheral or placental infection) was 16.8%, considerably lower than that of 23.2% reported in the neighbor district of Manhiça in 2009 [40], but is higher than the 6% reported among delivering women in the same area in 2012 [41]. In other African countries with stable transmission, the prevalence of infection at delivery ranged from 8.1 to 57.8% [42–45]. The major factor associated with infection at delivery in this study was living in rural areas. Rural villages in the area are at higher proximity to irrigation systems, which may provide additional breeding sites for mosquitoes increasing the overall risk of infection. In addition, IPTp-SP uptake is also lower in rural villages (associated with non-institutional deliveries) in concordance with other studies in Mozambique [46] and SSA [47, 48].

Although the effect of ≥ 3 doses of IPTp-SP in reducing maternal malaria infection at delivery was non-significant, there was a trend of decreasing parasite densities with increasing number of IPTp doses indicating a benefit of higher IPTp-SP uptake on reducing parasite density [34, 49].

Women at their first pregnancy and at younger age (< 20 years) were more likely to be infected at delivery, present higher parasite densities, and give birth to LBW and pre-term infants, compared to multigravidae and older women, respectively, confirming higher susceptibility to MiP and related adverse effects due to inadequate pregnancy-associated immunity [7, 50–53]. Moreover, young mothers may represent a particularly disadvantaged risk group characterized by low socioeconomic status and level of education, which may have an influence on health-related behavior [54]. Therefore, higher efforts to improve uptake of IPTp in this risk group should be stressed.

The majority of infections at delivery were sub-microscopic and asymptomatic (76.5%), which supports a role of asymptomatic pregnant women as malaria reservoir of infection and in contributing to the maintenance of malaria transmission [55, 56] (although gametocyte carriage in pregnant women should be measured to confirm this hypothesis). Sub-microscopic infections during pregnancy may have an harmful effect on the pregnant women and to the developing fetus [44, 57], however no association between infection and adverse pregnancy outcomes was observed. Although the study may have been unpowered to find significant associations in this regard, it is also well known that SP resistance affect IPTp-SP efficacy and thus, prevalence of sextuple mutated parasites in the study area should be evaluated [58, 59].

Placental malaria is associated with maternal and neonatal adverse outcomes in pregnancy [4, 5]. The proportion of placental infections by histology was 8.8%, the majority being past infections. The presence of PM was not correlated with peripheral infections, while similar results have been reported in other studies [52, 60].

Although IPTp-SP coverage may have been overestimated since the representation of the rural population in the study is lower than that in the general population of the district, and rural women are those at higher risk of low IPTp-SP uptake, the main strengths of this study was the enrollment of women in the community and health facilities including those with non-institutional deliveries, which allowed us to accurately investigate the factors affecting IPTp uptake under routine circumstances in women with different access to health facilities and thus to IPTp-SP.

## Conclusion

In conclusion, the study reports a IPTp-SP coverage for two and ≥ 3 doses of IPTp-SP higher than estimates from a HH survey in the same province, but still far below the national target of 80% coverage of ≥ 3 doses. Ongoing and new campaigns aiming to increase the use of malaria prevention strategies during pregnancy should particularly target rural populations, increasing IPTp knowledge, stimulate early visits to ANC and, importantly, improving access to health services and the quality of the services provided.

## Additional file

**Additional file 1: Table S1.** Characteristics of the study population by delivering place and associations with non-institutional delivery.

## Abbreviations

ANC: antenatal care visit
DOT: directly observed treatment
HDSS: health and demographic surveillance system
HH: household
IPTp: intermittent preventive treatment for pregnant women
ITN: insecticide-treated net
LBW: low birth weight
MiP: malaria in pregnancy
SP: sulfadoxine–pyrimethamine
SSA: sub-Saharan Africa
